# Clinical efficacy of Professional Continuous Glucose Monitoring in improving glycemic control among children with Type 1 Diabetes Mellitus: An Open-label Randomized Control Trial

**DOI:** 10.1038/s41598-019-42555-6

**Published:** 2019-04-16

**Authors:** K. V. Raviteja, Rakesh Kumar, Devi Dayal, Naresh Sachdeva

**Affiliations:** 1 Pediatric Endocrinology and Diabetes Division, Department of Pediatrics, Postgraduate Institute of Medical Education and Research (PGIMER), Sector 12, Chandigarh, India; 2 Department of Endocrinology, Postgraduate Institute of Medical Education and Research (PGIMER), Sector 12, Chandigarh, India

## Abstract

Frequent self-monitoring of blood glucose (SMBG) is the only accurate method available for insulin dose titration in patients with T1DM. Professional continuous glucose monitoring (p-CGM) is blinded recording of glucose trends over 5–7 days and helps physicians to guide insulin titration to patient. This study was planned to assess efficacy of insulin dose adjustments, based on p-CGM plus SMBG in improving glycemic control compared to SMBG alone. We did an open-label, parallel design, randomized control trial among children (2–10 years) having T1DM for at least 6 months. Subjects in the *intervention group* were placed on p-CGM (iPRO 2™ Professional CGM, Medtronic, USA) for 3–5 days along with regular SMBG. Data from p-CGM was analyzed by physician and used to guide insulin titration along with SMBG over following 3 months. *Control group* had only SMBG records for titrating insulin doses. Primary outcome was change in HbA1c 3 months after intervention. A total of 68 eligible children were randomized, 34 each to either arms. Thirty children in intervention group and 33 in control group completed the study and were analyzed. It was found that there was more decreased unit change in HbA1c, percentage of low sugar records and total insulin requirement per day, after 3 months follow-up, in intervention group. However, difference was not significant except for total insulin Units/kg/day (p = 0.014). In sub-group analysis of children with baseline HbA1c >7.5%, there was a significant mean fall of HbA1c by 1.27% (p = 0.045). There were no major adverse events associated with p-CGM. We conclude that addition of p-CGM along with SMBG may help in adjusting insulin dose more effectively especially in children with higher baseline HbA1c.

## Introduction

Multiple daily insulin injections (MDI) with frequent self-monitoring of blood glucose (SMBG) has been a standard of care in the management of Type 1 DM following results of the Diabetes Control and Complications Trial (DCCT)^1,2^. However, the major drawback with SMBG is poor compliance and even if frequently performed, it does not provide a complete picture of glucose trends. The factors like pain, inconvenience, and cost (in poor patients) contribute to poor compliance of SMBG. Despite active education and psychosocial support, only 4 to 6 glucose records per day are available through SMBG, which gives very limited data about the true glycemic profile. Further, it is well established that despite frequent SMBG and even a satisfactory HbA1c, a large number of hypoglycemic episodes (especially nocturnal and asymptomatic) and hyperglycemic episodes may remain undetected^3–6^. Thus, it is difficult to achieve optimal glycemic control, despite intensive insulin therapy and frequent self-monitoring of blood glucose.

To address these problems of SMBG, efforts have been made to develop accurate and feasible continuous glucose monitoring systems (p-CGM). With the use of p-CGM, it has become more apparent that conventional SMBG does not provide real trends of blood glucose levels and continuously recorded glucose data by p-CGM gives a better picture of glucose trends and thus can help guiding insulin doses more accurately.

Till date, only a few studies have been conducted to assess the efficacy of p-CGM in T1DM among pediatric population especially below 7 years of age. Of the 5 trials on professional p-CGM in children, 3 trials^7–9^ have shown small (but statistically significant) decrease in HbA1c in children undergoing p-CGM. However, other 2 trials^5,10^ have not shown any significant improvement in HbA1c with the use of p-CGM.

Systematic reviews of the available trials in children did not show clear-cut evidence on the efficacy of p-CGM in improving HbA1c^11–13^. All above evidence is based on a small number of subjects in each trial, largest trial in children^10^ included 36 children including controls. Further, there is limited data on p-CGM use in children with T1DM below 7 years.

We planned this study to assess the efficacy of insulin dose adjustments in improving glycemic control among children with T1DM, based on glucose data from combined p-CGM and SMBG when compared to SMBG alone.

## Methods

The study was conducted from Feb 2015 to Nov 2015 at a tertiary care pediatric hospital in North India. It was an open-label, parallel, randomized control trial registered with Clinical Trial Registry of India (Reg. No CTRI/2017/02/007937; Date of Registration 21 Feb 2017, accessible at www.ctri.nic.in). The details of study protocol and registration are provided in supplementary file. Children between 2 to 10 years with diagnosis of T1DM for at least 6 months, on the basal-bolus regimen with insulin analogs taking 3–4 injections per day and doing SMBG at least 3 times per day were included in the study. Children with known poor compliance and HbA1c >12% (107.7 mmol/mol), a history of DKA within previous 2 months or with associated co-morbidities like thyroid disorder and celiac disease were excluded. Hypothyroidism and Celiac disease were included in exclusion criteria (after protocol registration) as these could also affect outcomes.

Subjects were randomized to intervention (p-CGM + SMBG) or control (SMBG alone) groups using computer generated random number list. Group allocation was concealed from investigator and subjects using sealed opaque envelopes. Subjects in the Intervention group were placed on p-CGM (IPRO 2 Professional CGM, Medtronic, USA) for 3–5 days along with regular SMBG. Data from p-CGM and SMBG was analyzed and used to guide insulin titration over following 3 months. Control group had only SMBG data for guiding insulin titration. *Freestyle Optium*™ glucometer (Abbott) was used for SMBG. Professional CGM device were owned by the hospital and sensors for each patient were provided by the investigators. Study was approved by “Institute Ethics Committee (Intramural), Postgraduate Institute of Medical Education and Research, Chandigarh” and a written informed consent was obtained from parents/caregiver and assent from children before enrollment in the trial. Study was conducted as per Declaration of Helsinki regarding ethical conduct of research involving human subjects.

### Study procedures

At enrollment, basic demographic data of the study subjects, the average of recent two HbA1c, the percentage of low or high sugars in last 3 months, insulin dose being received etc was recorded in a predesigned proforma.

The p-CGM was placed and used as per user instructions provided by the manufacturer^14^. Subjects were encouraged to follow their regular lifestyle and treatment during monitoring period and were instructed to record their daily activity, meals, snacks and other relevant information with the exact time. Record of timing and doses of insulin injections was also maintained. Information with respect to duration, timing and problems in wearing of CGM, reasons for removing CGM if at all, any restriction in daily activities of the child (as told by parents/child) was also noted. Subjects continued to do SMBG at least 4 times per day pre-breakfast, pre-lunch, pre-dinner and bedtime and additionally during hypoglycemia symptoms were suspected, during and after p-CGM. CARELINK™ iPRO therapy management software for diabetes was used to upload CGM data and generate reports as per the instructions in the user guide^15^. Accuracy and reliability of p-CGM data were assessed using standard parameters available in the report generated by the software. P-CGM reports were analyzed objectively recognizing various patterns of glucose trends at various time frames in 24 hour period. Special attention was given to recognize Somogyi or Dawn phenomenon. Further modification in treatment was advised based on p-CGM and SMBG records.

In control group, no p-CGM was done and treatment advice/modifications were based only on SMBG records.

#### Procedures for insulin dose adjustments

Only two consultants in the clinic advised change in insulin doses for all patients. Standard principle of modification of insulin doses included looking at SMBG records of last 3 months (with more weightage given to recent 1 month) and identifying trends of glycemic patterns as observed on SMBG records. Specific insulin dose was increased or decreased if trend of the SMBG records following the dose showed higher or lower glucose respectively. If no trends were identified and SMBG showed fluctuating sugars stress was given to follow consistent diet and activity plan. Correction dose of insulin (ISF) and approximate carbohydrate ratio (ICR) were advised depending on total daily dose (TDD) of insulin using standard formulae. For the intervention group, P-CGM was performed around 1–2 weeks prior to the pre-decided date for clinic visit and P-CGM report was downloaded by the investigator and handed over to Consultant Incharge who analyzed the report of P-CGM and advised insulin dose and treatment modifications considering the glucose trends and information provided by the P-CGM in addition to SMBG records of past 3 months brought by patient on day of clinic visit.

HbA1c (primary outcome of the study), the percentage of high and low sugars over 3 months and insulin dose required (secondary outcome of the study) was recorded at baseline and again after 3 months follow-up post randomization in either of the study groups. A SMBG value of >200 mg/dl or <70 mg/dl was considered as high or low sugar respectively.

### Details of p-CGM used (iPRO 2™ CGM system, MMT-7745, medtronic, USA)

P-CGM measures glucose in interstitial fluid every 5–10 seconds through subcutaneous sensor, averages these values and stores a single value in the recorder every 5 min for upto 7 days. This system has been validated by several reports^16,17^ and was also shown to provide a very good correlation between blood and interstitial fluid glucose levels^18–20^. A minimum of 4 SMBG values from glucometer are required to calibrate the glucose sensor data provided in form of electrical signal in nano-Amperes (nA) to convert to glucose in mg/dl.

### Sample size

Calculated with a 1:1 randomization ratio and based on the assumption of a common SD of 1.5% and an absolute difference of 1% in HbA1c between study groups with an alpha error of 0.05 (two sided) and a beta error of 0.20, using formula n = 2 × K × SD^2^/delta^2^ (where K is constant, SD is standard deviation and delta is magnitude of difference to be detected). A sample size of 34 subjects each in the intervention and control group was calculated assuming no drop-outs for a 3-month short follow-up study.

### Statistical analysis

Mean (SD) or the Median (Interquartile range) and Range were calculated for the descriptive data. For changes in HbA1c, the percentage of low/high glucose records and insulin doses at two different study points, Paired t-test or Wilcoxon-Signed rank test was used as applicable. Differences between the two groups, as well as in sub-groups were examined using Independent sample T-test for quantitative tests and the Mann-Whitney U-test. P value < 0.05 was considered statistically significant with 95% confidence interval. Analysis was performed using SPSS statistical software version 20.0 (SPSS Inc., Chicago, IL, USA).

### Ethical approval

All procedures performed in studies involving human participants were in accordance with the ethical standards of the institutional research committee and with the 1964 Helsinki declaration and its later amendments or comparable ethical standards. This article *does not contain any studies with animals* performed by any of the authors.

### Informed consent

Informed consent was obtained from all individual participants included in the study.

## Results

**Figure 1:**
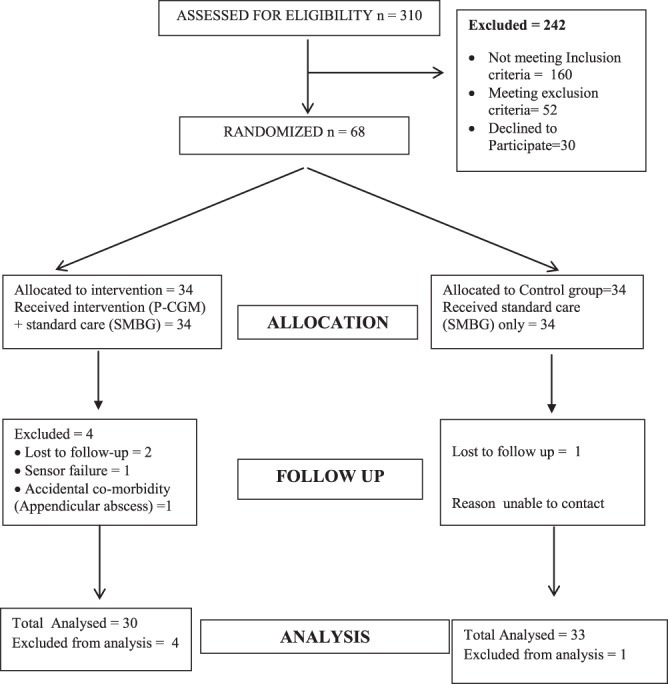
Study Flow Diagram.

Out of 310 children screened for eligibility from Feb 2015 to Nov 2015, a total of 68 subjects were enrolled in the study with 34 subjects in each group as shown in Fig. 1. However, 30 subjects in the intervention group and 33 in control group could be followed up until the end of the study (after 3 months) and were analyzed further. All baseline characteristics of study subjects were comparable between intervention and control groups as shown in Table 1.

**Table 1:** Baseline characteristics of subjects with T1DM in Intervention (P-CGM+SMBG) and Control (SMBG only) groups.

S.No	Baseline Parameters		P-CGM + SMBG n = 30	SMBG alone n = 33	P value
**1**.	Age (months) Mean ± SD		70.02 ± 25.30	75.26 ± 25.30	0.443
**2**.	Gender	Male	15 (50%)	19 (57.6%)	0.547
		Female	15 (50%)	14 (42.4%)	
**3**.	Duration of Diabetes (months) Median (IQR)		12 (8.5–24.25)	11 (6–22.5)	0.544*
**4**.	Average HbA1c (6 months) (%) (mmol/mol)		8.01 ± 1.46 (64 ± 2.9)	7.86 ± 1.18 (62.4 ± 2.1)	0.673
**5**.	Total insulin dose (U/Kg/Day)		1.12 ± 0.36	1.07 ± 0.38	0.627
**6**.	Percentage Hyperglycemic records^#^		21.82 ± 14.13	21.88 ± 16.36	0.989
**7**.	Percentage Hypoglycemic records^#^		7.88 ± 6.45	7.93 ± 5.32	0.690
**8**.	Anti-GAD 65 Antibody Positive		4 (13.3%)	9 (27.3%)	0.172

*Mann Whitney U test; ^#^Calculated from SMBG records of previous 3 months.

**Figure 2:**
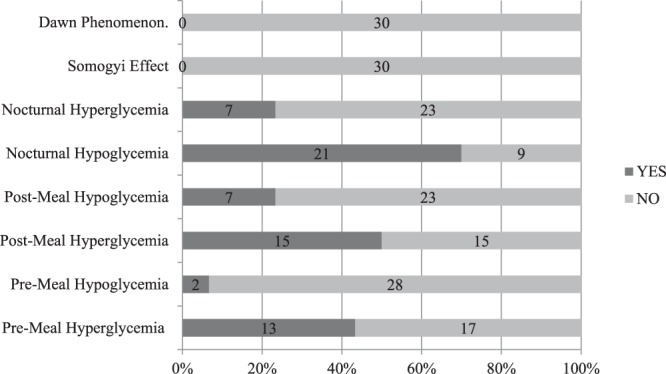
Findings of abnormal glucose trends as reported on P-CGM data (n = 30) in children with T1DM.

Various patterns of abnormal glucose trends reported on p-CGM in the intervention group are given in Fig. 2. Most common abnormal patterns noted were nocturnal hypoglycemia (21/30) followed by post meal hyperglycemia (15/30) and premeal hyperglycemia (13/30).

### Change in HbA1c (primary outcome) in two groups

In the intervention group, HbA1c (%) of the subjects decreased from 8.01 ± 1.46 (64 ± 2.9 mmol/mol) (mean ± SD) to 7.47 ± 0.91 (58 ± 1.8 mmol/mol) (mean ± SD) after 3 months of intervention (p = 0.05), whereas in control group this change was minimal from 7.86 ± 1.18% (62.4 ± 2.1 mmol/mol) to 7.85 ± 1.50% (62 ± 3 mmol/mol) (mean ± SD) (p = 0.92). Seventeen out of 30 (56.6%) subjects in the intervention group and 15 out of 33 (45.4%) subjects in control group achieved a decrease in HbA1c. However, the unit change in HbA1c was comparable in two groups (p = 0.145) as shown in Table 2.

**Table 2:** Comparison of difference in outcome parameters (over 3 months) in two groups with T1DM (per protocol analysis).

S.No	Change in Outcome Parameters (Baseline- at end of 3 month)	Intervention (n = 30) P-CGM + SMBG Mean ± SD	Control (n = 33) SMBG alone Mean ± SD	P value^a^
1.	Change in HbA1c (%)	0.54 ± 1.42	0.01 ± 1.41	0.145
2.	Change in % of hypoglycemic records^#^	0.38 ± 5.07	0.41 ± 6.66	0.897
3.	Change in % of hyperglycemic records^#^	1.91 ± 12.94	2.54 ± 12.45	0.845
4.	Change in Insulin dose (Units/Kg/day)	0.10 ± 0.18	−0.01 ± 0.16	**0.014**

^a^Independent sample T test; ^#^Calculated from SMBG records of previous 3 months.

In sub-group of subjects with HbA1c >7.5% (58 mmol/mol) at the baseline (36 out of 63), statistically significant fall in HbA1c % (1.27 ± 1.46 Vs 0.13 ± 1.76) was noticed in the intervention group compared to control group (**p** = **0.045)** as shown in Table 3. This difference was not significant in sub-group where HbA1c was less than 7.5% (58 mmol/mol) at the baseline.

**Table 3:** Difference in unit change of outcome parameters (over 3 months) in subjects with T1DM in two groups with baseline average HbA1c more than 7.5%(58 mmol/mol).

S.No	Change in Outcome Parameters (Baseline- at end of 3 month)	Intervention (n = 16) P-CGM + SMBG (Mean ± SD)	Control (n = 20) SMBG alone (Mean ± SD)	P^a^ value
1.	Change in HbA1c (%)	1.27 ± 1.46	0.13 ± 1.76	0.045
2.	Change in % of hypoglycemic record^#^	0.34 ± 6.09	2.16 ± 5.95	0.373
3.	Change in % of hyperglycemic record^#^	2.36 ± 10.45	5.34 ± 14.18	0.487
4.	Change in Total insulin (Units/Kg/day)	0.13 ± 2.18	0.05 ± 0.13	0.161

^a^Independent sample T test; ^#^Calculated from SMBG records of previous 3 months.

### Change in secondary outcomes

#### Change in percentage of Hypo and Hyperglycemic records in two groups

In the intervention group, baseline percentage of hypoglycemic records was 7.88 ± 6.45 (Mean ± SD) which decreased to 7.49 ± 6.89 (Mean ± SD) after 3 months and it decreased in 15 out of 30 (50%) subjects. In control group, baseline percentage of hypoglycemic records was 7.93 ± 5.32 (Mean ± SD) and in follow-up decreased to7.47 ± 6.87 (Mean ± SD) and fall was observed in 21out of 33 (63.6%) subjects. However, this change in percent of hypoglycemic records from baseline to 3 months was comparable in two groups (p = 0.897).

Baseline percentage of hyperglycemic records in the intervention group was 21.82 ± 14.13 (Mean ± SD) which decreased to 19.91 ± 10.77 (Mean ± SD) after 3 months and corresponding values in control group were 21.88 ± 16.36 and 19.34 ± 11.44. Sixteen (53.3%) subjects in the intervention group and 17 (51.1%) subjects in control group achieved a decrease in the percentage of hyperglycemic records over 3 months of follow-up. Again, the change was comparable in two groups, (p = 0.845) as shown in Table 2.

#### Change in total insulin dose (Units/kg/day) in two groups

In the intervention group, baseline insulin requirement was 1.12 ± 0.36 U/kg/day and at follow-up it decreased to 1.01 ± 0.33 U/kg/day. These values in the control group were 1.07 ± 0.38 U/kg/day and 1.08 ± 0.38 U/kg/day (Mean ± SD) respectively. Twenty-one (70%) subjects in the intervention group and 18 (54.5%) in control group achieved a decrease in total insulin dose over 3 months of follow-up. The decrease in total insulin units was significantly more in intervention group when compared to control group **(p** = **0.014)** as shown in Table 2.

The overall attrition rate in our study was 7.94% as 5 subjects did not complete follow-up after randomization into either of the treatment arms and were excluded from per-protocol analysis. Of these subjects who did not complete study, 1 subject was in control group and 4in the intervention group. So, attrition rate in the intervention group was 8.82% and in control group, it was 2.94% with a differential attrition rate of 5.88%.

### Intention to treat (ITT) analysis

Out of 68 enrolled subjects randomized into two groups, 4 subjects in the intervention group and 1 in control group did not complete follow-up. Thus, 63 subjects were followed until end of the study and analyzed. In these 5 subjects who were lost to follow-up, follow-up outcome parameters of HbA1c, hypoglycemic records percent, hyperglycemic records percent and total insulin dose were presumed to be same as at baseline (at enrollment). With this assumption based on the principle of simplest imputation method of “Last Observations Carried Forward [LOCF]”, Intention-to-treat analysis was performed for all 68 subjects. The change in outcome parameters as analyzed by ITT analysis was similar to the results of the per-protocol analysis as mentioned in Table 2.

### Adverse events and technical problems with p-CGM

The average duration of p-CGM recording was 87.5 hours. Major adverse effects like swelling and bleeding at the local site were not noticed in any of the 30 subjects analyzed. A significant number of subjects (n = 10, 33.3%) experienced minor problems of redness at the site of sensor insertion while local irritation in 12 (40%) subjects and parental apprehension was reported in 11 (36.6%) subjects. Premature and accidental removal of CGM occurred in 2 subjects out of 6 (20%) subjects who reported fixing and stability problem.

A total of 39 sensors (a disposable component of the p-CGM) were used for 34 subjects enrolled in the study out of which 3 subjects underwent repeat p-CGM due to recording duration of fewer than 48 hours with first sensor insertion. Two sensors were damaged and discarded as they could not be inserted properly in the first attempt.

### p-CGM data quality

Minimum of 2 calibrated paired meter glucose readings per day which is required for acceptable data quality of CGM records was seen in all 30 subjects analyzed. Other parameters for acceptable data quality; Mean Absolute Difference (MAD) of <28% and Correlation Coefficient of >70% was observed in 24 (80%) subjects each.

## Discussion

In this RCT, we studied the efficacy of insulin dose adjustments based on data obtained from p-CGM along with SMBG in improving glycemic control in 68 subjects with T1DM on MDI regimen, compared to SMBG alone. The baseline characteristics were balanced in the two groups.

There was no significant difference observed in the unit change in HbA1c between two groups after 3 months of intervention. However, in the subgroup with HbA1c more than 7.5% at baseline, we found a significant difference in unit change in HbA1c in two groups. There was a decrease in the percentage of both hypoglycemic and hyperglycemic records (as seen on SMBG) in both the groups over a 3-month post-intervention period, however, the difference was not statistically significant. The only significant difference observed was in unit change (delta change) of insulin dose requirement (U/kg/day). The decrease in insulin dose was more in the intervention group as compared to control group.

Results of our study were consistent with some of the previous randomized control studies on p-CGM in children with T1DM^5,10^ and latest systematic reviews where no clinically significant change in HbA1c was detected in patients who underwent p-CGM in addition to SMBG^11,12^.

Our study was powered to detect a difference of more than 1% of HbA1c in 2 groups as significant. Whereas most of the previous studies^7–9^ which have shown a significant benefit of p-CGM in improving HbA1c, the actual fall in HbA1c was to the tune of 0.5–1%. Also, in a recent retrospective analysis of 122 youth with T1DM who underwent p-CGM for various reasons, it was seen that only 32% had >0.5% decrease in HbA1c following p-CGM^21^. Further, above quoted studies were different from our study as they had a small sample size and they used p-CGM repeatedly over 1–6 months of the study period.

Of the few studies showing significant benefit of p-CGM on HbA1c reduction, a pilot study by Chase *et al*.^7^, on eleven subjects (10–17 years) with type 1 diabetes having average HbA1c values >8.0%, showed difference of only 0.36 ± 0.07% after p-CGM done every month for 3 months in intervention group, whereas we observed a higher magnitude of reduction in HbA1c which was significant in subjects with baseline HbA1c >7.5% (58 mmol/mol). In another controlled cross-over trial by Ludvigsson *et al*.^8^ where p-CGM in 27 diabetic patients aged 5 to 19 years was done every 2 weeks over 6 months in open and blinded arms which were crossed over in midway of the study, HbA1c decreased from 7.70% to 7.31% in the open arm which is comparable to our findings.

In a single-blind RCT by Lagarde *et al*.^9^ including 27 children, the decrease in HbA1c of 0.61 ± 0.68% in the intervention group was statistically significant (p = 0.03) when compared to controls. We also observed a similar magnitude of change in HbA1c in the intervention group but it was not significantly different from the control group.

Two systematic reviews^13,22^ including studies both on p-CGM and real-time CGM to assess the efficacy of CGM in improving glycemic control showed that use of CGM is associated with improvement in metabolic control in T1DM, with significant short- and long-term reductions in HbA1c and reduction in the duration of periods of hypoglycemia and hyperglycemia. But, the inclusion of studies using real-time CGM in their meta-analysis would make it difficult to make any conclusions on the efficacy of p-CGM alone in reducing HbA1c.

Our study was appropriately designed with sufficient sample size with appropriate duration and quality of p-CGM, still we could not find any significant difference in HbA1c at three months following intervention. This suggests that p-CGM done over 3–5 days may apparently give information on glucose trends and help in guiding treatment in children with T1DM, but it does not reflect into improved HbA1c. Another explanation of no significant difference between the two study groups could be the “study effect” itself which made subjects in both the groups more compliant to multidisciplinary management and hence a similar degree of decrease in HbA1c. Better glycemic control in pediatric population demands parents and family co-operation. Most recognized hindrance to good glycemic control in children with type 1 DM is poor adherence to multi-disciplinary diabetic management due to various reasons. Despite the availability of more detailed information on 24-hour glucose trends from CGM and making more judicious and wiser adjustments in insulin doses, dietary modifications and other changes in daily activities, it did not reflect in significantly better glucose control in the intervention group.

On subgroup analysis of children with HbA1c more than 7.5% at baseline, we found a significant difference in change in HbA1c in two treatment groups (mean fall of 1.27% versus 0.13%). Although it was a posthoc analysis of the subgroup of subjects (36 out of 68), this information suggests that p-CGM could be particularly useful in sub-group of patients with poorly controlled type 1 DM and high HbA1c. However, this significant result could also indicate phenomenon of regression to mean. Further, it is well accepted that it is difficult to improve the HbA1c when it is already in the higher side but within the target range; and relatively easier to get a higher unit fall in HbA1c when it is high above the target range. The HbA1c (glycemic control over previous 3 months) depends on a number of factors including dietary compliance, activity, age of the patients, diabetes self-management skills of the parents etc. So, assessment of all these variables is important to attribute the improved glycemic control to p-CGM alone. To minimize the effect of all these factors/variables, we planned randomized control trial where patients with similar baseline characteristics were analyzed 3 months after the intervention with the assumption that all these variables (affecting HbA1c) will not change significantly during 3-months observation period. In sub-group with less than 7.5% HbA1c at baseline, there was no significant difference in outcome variables in follow-up. Overall, in all patients included (regardless of HbA1c at baseline) there was fall in HbA1c in the group who underwent P-CGM when compared to SMBG alone group (0.54 versus 0.01%). A fall of 0.5% in HbA1c seems clinically significant but did not reach statistical significance as sample size was calculated for detecting 1% change in HbA1c.

A significant difference in two groups in the decrease of total insulin requirement (U/kg/day) suggests that p-CGM may be useful in optimizing insulin dose required. However, this may not be of clinical significance and have any implication for metabolic control. Reduction in insulin dose after p-CGM could be explained by more judicious use of insulin dosage with more detailed information on glucose trends available on CGM records.

The attrition rate in our study was 7.94% with a differential attrition rate of 5.88%. When this overall attrition rate and differential attrition rate were plotted on cut-offs for What Works Clearinghouse (WWC) attrition standards, our study was in the area under low level of attrition^23^. To see the effect of attrition on our study results we have also done Intention to treat (ITT) analysis with the help of simple imputation, based on the principle of Last Observations Carried Forward (LOCF). Both primary and secondary outcomes in ITT analysis were similar to the results of the per-protocol analysis. Another limitation of the study was that a significant number of enrolled patients were within 1 year of diagnosis, when honeymoon phase may interfere with assessing insulin dosing and effect of intervention on glycemic control. The median age in intervention group was 12 months and 11 months in control group with youngest patient enrolled being 8 months and 6.5 months respectively, in two groups. Some patients in the control group could have been still in the honeymoon phase or just coming out from it and thus affecting HbA1c and insulin dose adjustments. Nocturnal hypoglycemia/hyperglycemia, an important determinant of glycemic control and its detection is a common indication for doing P-CGM, could not be assessed after three months of intervention as we could not repeat P-CGM after three months due to financial constraints.

The study design was the main strength of our study and all possible confounding factors were balanced in the two groups. To the best of our knowledge, this is the largest RCT assessing efficacy of p-CGM in improving HbA1c in children, till date. Also, this is the first study from a developing country like India. Apart from HbA1c we also studied other important parameters of the frequency of low and high sugar records, pre and post intervention. Glucose variability has also been recently recognized as a marker of poor diabetes control leading to long-term complications. These parameters were not seen in some of the previous studies^6,7^.

Our study results can be generalized to all type 1 DM children on basal-bolus regimen except those with very poor glucose control (>12% HbA1c) as they were excluded due to very poor compliance to SMBG and other treatment recommendations. Despite some reservations about the use of p-CGM in children less than 7 years, our study has amply shown that it is feasible and acceptable even in younger children. There were no significant adverse events noted and no significant technical failures when using it in younger children. The quality of p-CGM data as per the standard parameters was also acceptable.

## Conclusions

P-CGM may be useful in certain groups of patients (like those with high HbA1c) with T1DM. Addition of intermittent p-CGM to daily SMBG may help understand continuous glucose trends over days and guide therapeutic modulation in children with T1DM, especially for those with high HbA1c. Further, P-CGM is safe and acceptable in children as young as 2 years old.

## Supplementary information

Study Protocol

Registration of Trial
