# Supplementary material for: Clinical efficacy of Professional Continuous Glucose Monitoring in improving glycemic control among children with Type 1 Diabetes Mellitus: An Open-label Randomized Control Trial
Source: Sci Rep. 2019 Apr 16;9:6120. doi: 10.1038/s41598-019-42555-6 (PMC6467868; doi:10.1038/s41598-019-42555-6)
Supplement: Supplementary file 1 — Study Protocol [file 41598_2019_42555_MOESM1_ESM.docx]

**Thesis protocol**

**Professional Continuous Glucose Monitoring and Self-Monitoring Of Blood Glucose in Children with Type 1 Diabetes Mellitus: An Open Label Randomised Control Trial**

Submitted in partial fulfilment of the requirements for the degree of

MD (Pediatrics)

of the

Postgraduate Institute of Medical Education and Research

Chandigarh.

by

**DR. K V RAVI TEJA**

Junior Resident, Pediatrics,

PGIMER, Chandigarh.

**GUIDE**

**DR. RAKESH KUMAR**

Assistant Professor

Department of Paediatrics,PGIMER. Chandigarh.

**CO-GUIDES**

**DR. DEVI DAYAL DR. NARESH SACHDEVA**

Associate Professor Assistant Professor

Department of Paediatrics, Department of Endocrinology

PGIMER, Chandigarh PGIMER, Chandigarh

**ABBREVIATIONS:**

AACE = American Association of Clinical Endocrinologists;

CGM = continuous glucose monitoring;

CPT = Current Procedural Terminology;

CSII = continuous subcutaneous insulin infusion;

DM = diabetes mellitus;

FDA = US Food and Drug Administration;

HbA1c = glycated hemoglobin;

JDRF = Juvenile Diabetes Research Foundation;

SMBG = self-monitoring of blood glucose.

BGL = blood glucose levels.

**INTRODUCTION**

A continuous glucose monitoring system (CGMS) records data obtained from a subcutaneous sensor every 5 min for up to 72 hr and provides the clinician with a continuous profile of tissue glucose levels. The interstitial glucose levels lag approximately 15 min behind the blood glucose values at any given level. The CGMS values tend to have a high correlation coefficient for blood glucose values ranging between 40 and 400 mg/dL.

CGMS is minimally invasive and entails the placement of a small, subcutaneous catheter that can be easily worn by adults and children. CGM can be used in two clinical scenarios. First, being **retrospective(professional CGM)** recording of glucose, usually with integrated insulin infusion pump, for 48-72 hours for clinicians to analyze and further make recommendations for treatment to patients based on trends seen over those 48-72 hours. The system provides information that allows the patient and health care team to adjust the insulin regimen and the nutrition plan to improve glycemic control. CGMS can be helpful in detecting asymptomatic nocturnal hypoglycemia as well as in lowering HbA_1c_ values without increasing the risk for severe hypoglycemia.

Second, use is **real time continuous glucose monitoring (personal CGM)** usually with integrated insulin infusion pump, which helps patient have real time knowledge of his blood sugar values and their trends based on which patient can take action as regard to insulin dose. While there are potential pitfalls in CGMS use, including suboptimal compliance, human error, incorrect technique, and sensor failure, the implementation of CGMS in ambulatory diabetes practice allows the clinician to diagnose abnormal glycemic patterns in a more precise manner.

**Professional CGM**: Continuous glucose monitoring equipment is attached to patients with their consent and they remain unaware of monitoring results until they are downloaded and analyzed. Professional continuous glucosemonitoring has similarities to other methods of retrospective monitoring in medicine and has been called the “Holter-monitor of diabetes”.

**Personal CGM**: In personal real-time CGM, glucose values are visible continuously to the patient; this allows for immediate therapeutic adjustments based on “real-time” glucose results. It is usually used along with insulin pumps.

**Advantages of Professional Continuous glucose monitoring compared to Real time CGM:**

1. Patient behavior is not influenced, so results are unbiased and can be used for therapeutic recommendations, including changes in diet, activity and medications.
2. Easier for patients to use and requires minimal training and set up**.**
3. It is cheaper compared to real-time CGM.

**Indications for professional continuous glucose monitoring in children with Type 1 DM(1,2):**

1. Children undergoing change in their diabetes regimen.
2. Nocturnal hypoglycemia/dawn phenomenon.
3. Hypoglycemia unawareness.
4. Postprandial hyperglycemia.
5. Patients with discrepancies between HbA1c and SMBG.
6. Patients unable to achieve goal with SMBG.

**WHY THIS STUDY?**

It is well known that tight glycemic control delays long-term complications of type 1 DM (3). Frequent monitoring of blood glucose by finger pricks with a glucometer by self/parents is conventionally done to see glucose control and insulin dose adjustments. However, self-monitoring of blood glucose (SMBG) done 4 times a day (as recommended) does not give idea of glucose trends over 24 hours.

Continuous glucose monitors are being used in the west for over a decade now to know the glucose trends over 24 hours. Professional CGM provides blood glucose values every 5 mins and is usually placed over 72 hours to see glucose tends over 3 consecutive days. Its accuracy and reliability has been established in adults. But till now only a few studies have been done on professional CGM in paediatric population especially in children below 8 years of years of age. Further, there are conflicting results regarding the beneficial effect of professional CGM in children as seen in few studies previously done (4, 5, 6, 7). Role of Professional CGMS in reducing HbA1c in Indian children with Type 1 Diabetes has not been studied. It is important to know that such a costly intervention should be recommended in Indian children as it may further add to financial burden in these patients.

**REVIEW OF LITERATURE**

There are previously very few studies done on use of professional CGM to demonstrate its efficacy in tight glycemic control and in reducing HbA1c levels. These are summarized in following table 1. Studies done on utility of Professional CGMS in adults and pregnant women are summarised in Table 2 and 3 respectively.

**Evidence for use of Real Time CGMS in Type 1 Diabetes**

1. A Cochrane review on “Continuous glucose monitoring systems for type 1 diabetes mellitus”,to assess the effects of CGM systems compared to conventional self-monitoring of blood glucose (SMBG) in patients with diabetes mellitus type 1 has been recently published. Twenty-two RCTs meeting the inclusion criteria of this review were identified. The results of the meta-analyses (across all age groups) indicate benefit of CGM for patients starting on CGM sensor augmented insulin pump therapy compared to patients using multiple daily injections of insulin (MDI) and standard monitoring blood glucose (SMBG). After six months there was a significant larger decline in HbA1c level for real-time CGM users starting insulin pump therapy compared to patients using MDI and SMBG (mean difference (MD) in change in HbA1c level -0.7%, 95% confidence interval (CI) -0.8% to - 0.5%, 2 RCTs, 562 patients, I2=84%). The risk of hypoglycaemia was increased for CGM users, but CIs were wide and included unity (4/43 versus 1/35; RR 3.26, 95% CI 0.38 to 27.82 and 21/247 versus 17/248; RR 1.24, 95% CI 0.67 to 2.29).

For patients starting with CGM only, the average decline in HbA1c level six months after baseline was also statistically significantly larger for CGM users compared to SMBG users, but much smaller than for patients starting using an insulin pump and CGM at the same time (MD change in HbA1c level -0.2%, 95% CI -0.4% to -0.1%, 6 RCTs, 963 patients, I2=55%). On average, there was no significant difference in risk of severe hypoglycaemia or ketoacidosis between CGM and SMBG users. The confidence interval however, was wide and included a decreased as well as an increased risk for CGM users compared to the control group (severe hypoglycaemia:36/411 versus 33/407; RR 1.02, 95% CI 0.65 to 1.62, 4 RCTs, I_2_=0% and ketoacidosis: 8/411 versus 8/407; RR 0.94, 95% CI 0.36 to 2.40, 4 RCTs, I_2_=0%).

**Table 1 - Studies previously done on PROFESSIONAL CGM in children with type 1 Diabetes:**

| First author, year | Patients | Baseline HbA1c | Age group years | % male | No.  CGM | No. Control | CGM duration of use | CGM  frequency  of use | SMBG description | Type of study | Results | Conclusion |
| --- | --- | --- | --- | --- | --- | --- | --- | --- | --- | --- | --- | --- |
| Yates, 2006(4) | Children, T1DM | 8.2% CGM  7.9% Control | 13-15 | 36 | 19 | 17 | 3 days | 3 days  every 3  weeks for  a 3 month  period | Four to six times daily finger stick testing | Randomised control study | there was no difference in the degree of improvement in A1C at 12 weeks betweenthe CGMS (-0.4% [95% CI -0.7 to -0.1]) and the control group (-0.4% [-0.8 to 0.2]). | CGMS was no more useful than intermittent finger stick SMBG. |
| Lagarde, 2006(8) | Children, T1DM | 8.4% interven-  tion  8.8% control | 7-17 | 44 | 18 | 9 | 3 days | At 0, 2,  and 4  months | Finger stick with BD logic 4 times/day glucometer with once weekly measure at 2 AM | Single blind randomised control trial | The decrease in HbA1c of (0.61 +_ 0.68%) in the  intervention group was statistically significant (p = 0.03), whereas the  decrease in HbA1c of (0.28 +_ 0.78%) in the control group.was not. | Use of CGMS improves metabolic control in children with type 1 DM. |
| Deiss, 2006(9) | Children, T1DM | Arm A 7.8%  Arm B8.4% | 7 - 11 | 53 | 15 | 15 | 3 days | Twice  (once  blinded,  once  unblinded) | Accu-Chek with finger sticks at least five times daily | Randomised controlied cross-over trial | No significant change of HbA1c occurred within each arm (A,p = 0.183 and B, p = 0.823), rrespectively whether CGMS datawere blinded or not. Likewise, mean glucose and hyper- or hypoglycaemiavalues did not change significantly. | use of CGMS in children with  type 1 diabetes did not result in a significant improvement of  metabolic control. |
| Ludvigson, 2003  (10) | Children, T1DM | 7.7% CGM, 7.75% control | 9-15 | Not provided | Total 27 (cross over study) | Total 27 (crossover study) | 3 days | 3 days  every 2  weeks | SMBG at least two times in a day and seven times in a day once a week | A controlled cross-over study | HbA1c decreased significantly in open-arm (from 7.70%-7.31%)but not in blind arm (7.75-7.65%). | CGMS a useful tool for education and improving metabolic control. |
| Chase, 2001(11) | Children, T1DM | 10% CGM  9.0% control | 10-17 | 55 | 5 | 6 | 3 days | Anytime  within the  30- day  Period | 4 times/day | Randomised control trial | CGMS, the 5 children had a significantly lower mean  HbA1c value compared with their initial value (mean 6standard error of the mean [SEM] decrease 5 (.36% +-.07%). The mean decrease for the controls was (.2%+-.2%). | CGMS allows recognition of nocturnal hypoglycaemia. |

**Table 2. Randomized Controlled Clinical Trials Evaluating the Effects of Continuous Glucose Monitoring in the Treatment of Type 1 Diabetes Mellitus**

|  | **Trial Name** | **Description** | | **Outcomes** | |
| --- | --- | --- | --- | --- | --- |
| Adults and adolescent | **STAR-1 (12)** | Primary end point: HbA1c  change from baseline  Also evaluated  hyperglycemia and/or  hypoglycemia incidence  Evaluated CSII patients 12 to  72 years of age | | 6-Month HbA1c (vs baseline HbA1c 8.44%)  CGM + SMBG-treated patients: 7.77%  SMBG patients: 7.84%  Patients with ≥60% sensor utilization compliance experienced  significant HbA1c reduction compared with less-compliant  patients (*P*<.05)  Severe hypoglycemia rates were higher in the CGM group^a^ | |
|  | **JDRF(13)** | Primary end point: HbA1c  change from baseline in CSII  and MDI patients  Also evaluated hypoglycemia  incidence  Evaluated patients 15 to 24  and ≥25 years of age (adult  groups) | | 26-Week HbA1c (vs baseline):  Age 15 to 24 years (baseline HbA1c 7.9%-8.0%)  Mean HbA1c difference of 0.08% for CGM + SMBG  patients vs SMBG alone^b^  Age ≥25 years (baseline HbA1c 7.6%)  Mean HbA1c difference of – 0.53% for CGM + SMBG  patients vs SMBG alone^a^  Severe hypoglycemic events were rare and occurred at the same rate for both study groups; both study groups also demonstrated similar biochemical hypoglycemia rates  Patients aged ≥25 years showed increased sensor use compared with other patients  Frequency of CGM monitoring was associated with  significantly greater HbA1c reductions in all study groups | |
| ***Key takeaway: More consistent CGM use predicts HbA1c reductions*** | | | | | |
| Youth | **DirecNet(14)** | | Two, 13-week pilot studies  (DirecNet); randomized  clinical trial (JDRF CGM)  Primary end point: HbA1c  change  Safety end point:  Hypoglycemia incidence | | HbA1c among CSII users improved from 7.1% at baseline to  6.8% at 13 weeks^a^  Hypoglycemia frequency changed from 4.5% at baseline to  5.5% at 13 weeks^b^ |
|  | **JDRF CGM(13)** | |  |  | After 26 weeks, HbA1c levels <7% in 27% of CSII users vs 12%  of control group (age 8-14 years)^a^  Patients who used the sensor 6 to 7 days a week were able to  lower their HbA1c level by a mean of 0.8% and maintain this  improvement for 12 months  Hypoglycemia rates did not differ between treatment groups |
| ***Key takeaway: Youth were much less likely than adults to use CGM devices on a near-daily basis; the best HbA1c-lowering***  ***results were seen in patients who used the sensor 6 to 7 days a week*** | | | | | |

Abbreviations: CGM, continuous glucose monitoring; CSII continuous subcutaneous insulin infusion; DirecNet, Diabetes Research in Children Network; HbA1c, hemoglobin A1c; JDRF, Juvenile Diabetes Research Foundation Sensor Study; MDI, multiple daily injections; SMGB, self-monitoring of blood glucose; STAR-1, Sensor-augmented pump Therapy for A1C Reduction.

a Statistically significant.

b Not significant.

**Table 3.Studies evaluating the efficacy of professional continuous glucose monitoring in pregnant women with diabetes mellitus.**

| Study | Goal | Patients | Duration | Intervention | Outcomes |
| --- | --- | --- | --- | --- | --- |
| Jovanovič  (2000) (15) | Evaluate professional CGM  to detect previous unknown  hyperglycemia in women  with GDM | 10 women with GDM  (no gestational data  provided) | 72 hours | Professional CGM | Mean total min/24 h previously  undetected hyperglycemia:  ~30 min |
| Yogev et al  (2003) (16) | Comparison of daily  glycemic profiles in  pregnant women with type  1 DM measured by  professional CGM vs  intermittent glucose  monitoring | 34 pregnant women with  type 1 DM, gestational  age 16 to 32 weeks,  receiving multiple  insulin injections | 72 hours | Professional CGM  Vsfingerstick  glucose  measurements  performed 6 to 8  times a day | Average of 780 ± 54 glucose  measurements recorded for  CGM patients; mean total  hyperglycemia in professional  CGM arm (undetected by  fingerstick): 192 ± 28 min/24  h; nocturnal hypoglycemic  events recordedin a total of 26  patients |
| Chen et al  (2003) (17) | Evaluate daily glucose level  in pregnant women with  GDM using professional  CGM vs SMBG | 57 women with GDM,  gestational age 24 to  35 weeks; 23 treated  by diet alone, 34 by  diet and insulin | 30 days | Professional CGM  vs SMBG with  fingerstick | Average of 763 ± 62 glucose  measurements recorded for  CGM patients; mean total  hyperglycemia (undetected by  fingerstick): 132 ± 31 min/24 h  in insulin-treated group and 94  ± 23 min/24 h in diet-treated  group; 14 patients, all insulin treated,  experienced nocturnal  hypoglycaemia |
| Murphy et al  (2008) (18) | Evaluate the effectiveness of  professional CGM during  pregnancy on maternal  glycemic control, infant  birth weight, and risk  of infant macrosomia in  women with type 1 DM  and type 2 DM | 46 women with type 1  DM and 25 women  with type 2 DM,  gestational age 8 to  32 weeks | 3 years | Antenatal care  plus professional  CGM (n = 38)  or standard  antenatal  care (n = 33);  professional  CGM offered for  ≤7 days every 4  to 6 weeks | Patients using professional CGM  had lower mean hemoglobin  A1c levels (5.8% vs 6.4%);  infants of CGM-using women  had decreased median birth  weight percentiles (69% vs  93%) and a reduced risk of  macrosomia (odds ratio 0.36;  95% CI, 0.13-0.98; *P* = .05) |

Health-related quality of life was reported in five of the 22 studies. In none of these studies a significant difference between CGM and SMBG was found. Diabetes complications, death and costs were not measured. Authors concluded that there is limited evidence for the effectiveness of real-time continuous glucose monitoring (CGM) use in children, adults and patients with poorly controlled diabetes. The largest improvements in glycaemic control were seen for sensor-augmented insulin pump therapy in patients with poorly controlled diabetes who had not used an insulin pump before. The risk of severe hypoglycaemia or ketoacidosis was not significantly increased for CGM users, but as these events occurred infrequent these results have to be interpreted cautiously.There are indications that higher compliance of wearing the CGM device improves glycosylated haemoglobin A1c level (HbA1c) to a larger extent (19).

2. Meta- analysis was conducted by Szypowska A et al, to explore the potential beneficial effects of the use of RT-CGM on diabetes management compared with self-blood glucose measurement (SBGM) in patients with type 1 diabetes. Seven RCTs (total patients- 948) met their inclusion criteria. Meta- analysis showed better HbA1c reduction in subjects using RT-CGM compared with those using SBGM (mean difference (MD) being - 0.25;95%confidence interval (95%CI): from -0.34 to -0.17;P<0.001).Patients treated with insulin pump and RT-CGM had a lower HbA1c level compared with subjects managed with insulin pump and SBGM (four RCTs, n =497; MD - 0.26; 95% CI: from -0.43 to -0.10; P<0.002). The benefits of applying RT-CGM were not associated with an increasing rate of major hypoglycemic episodes. The use of RT-CGM for over 60–70% of time was associated with a significant lowering of HbA1c.Authors concluded that RT-CGM is more beneficial than SBGM in reducing HbA1c in patients with type 1 diabetes. However, suggested that further studies are needed to evaluate the efficacy of this system in the pediatric population, especially in very young children as number of children in studies analysed was very less (20).

3**.** Another meta-analysis by Golden SH et al including 41 RCTs (including adults and children with either type1 or type 2 DM) showed that in children and adults with type 1 diabetes, CSII use was associated with improved quality of life compared with MDI (low SOE). They found studies of the comparative effectiveness of RT-CGM versus SMBG in individuals with type 1 diabetes only. Compared with SMBG, RT-CGM achieved a lower HbA1c, with greater reductions occurring where sensor compliance was 60 percent or greater (high SOE). There was no difference in the rate of severe hypoglycemia (low SOE) or quality of life (low SOE).They concludedthat RT-CGM is superior to SMBG in lowering HbA1c, without affecting the risk of severe hypoglycemia, in individuals with type 1 diabetes, particularly when compliance is high. Sensor-augmented pumps are superior to MDI/SMBG in lowering HbA1c (21).

4. In a meta-analysis to review how intensive insulin therapy (multiple daily injections [MDI] vs. rapid-acting analogue -based continuous subcutaneous insulin infusion [CSII]) or method of monitoring (self-monitoring of blood glucose [SMBG] vs. real-time continuous glucose monitoring [rt-CGM]) affects outcomes in type 1 and 2 diabetes mellitus, 33 randomized controlled trials in children or adults that compared CSII with MDI (n = 19), RT-CGM with SMBG (n = 10), or sensor-augmented insulin pump use with MDI and SMBG (n = 4) were included of analysis. Results revealed that MDI and CSII had similar effects on hemoglobin A1c (HbA1c) levels and severe hypoglycemia in children or adults with type 1 diabetes .Compared with SMBG, RT-CGM achieved a lower HbA1c level (between-group difference of change, -0.26% [95% CI, -0.33% to -0.19%]) without any difference in severe hypoglycemia. Sensor-augmented insulin pump use decreased HbA1c levels more than MDI and SMBG did in persons with type 1 diabetes mellitus (between-group difference of change, -0.68% [CI, -0.81% to -0.54%]). Authors, thus, concluded that continuous subcutaneous insulin infusion and MDI have similar effects on glycemic control and hypoglycemia, except CSII has a favourable effect on glycemic control in adults with type 1 diabetes mellitus. For glycemic control, rt-CGM is superior to SMBG and sensor-augmented insulin pumps are superior to MDI and SMBG without increasing the risk for hypoglycemia (22).

5. In another meta-analysis to determine the clinical effectiveness of real time continuous glucose monitoring compared with self-monitoring of blood glucose in type1diabetes.Six trials were identified, consisting of 449 patients randomized to continuous glucose monitoring and 443 to self-monitoring of blood glucose. The overall mean difference in HbA1c for continuous glucose monitoring versus self-monitoring of blood glucose was −0.30% (95% confidence interval −0.43% to −0.17%) (−3.0, −4.3 to −1.7 mmol/mol).Continuous glucose monitoring was associated with a significant reduction in HbA1c percentage, which was greatest in those with the highest HbA1c at baseline and who most frequently used the sensors. Exposure to hypoglycaemia was also reduced during continuous glucose monitoring. The most cost effective or appropriate use of continuous glucose monitoring is likely to be when targeted at people with type 1 diabetes who have continued poor control during intensified insulin therapy and who frequently use continuous glucose monitoring (23).

6. Another meta-analysis of 19 trials to assess the efficacy of continuous glucose monitoring (CGM) in improving glycemic control and reducing hypoglycemia compared to self-monitored blood glucose (SMBG), showed that CGM was associated with a significant reduction in mean hemoglobin A1c [HbA1c; weighted mean difference (WMD) of -0.27% (95% confidence interval [CI] -0.44 to -0.10)]. This was true for adults with T1DM as well as T2DM [WMD -0.50% (95% CI -0.69 to -0.30) and -0.70 (95% CI, -1.14 to -0.27), respectively]. No significant effect was noted in children and adolescents. There was no significant difference in HbA1c reduction between studies of real-time versus non-realtime devices (WMD -0.22%, 95% CI, -0.59 to 0.15 versus -0.30%, 95% CI, -0.49 to -0.10; p for interaction 0.71) (24).

**AIMS AND OBJECTIVES**

**Primary objective:**

1. To assess efficacy of professional CGM (done over 3 days) along with self-monitoring blood glucose (SMBG) in improving glycemic control compared to SMBG alone.

**Secondary Objectives:**

1. To assess feasibility and acceptability of professional CGM in diabetic children between 2 to 8 years of age.

2. Efficacy of professional CGM in reducing hypoglycemic and hyperglycemic episodes.

**RESEARCH QUESTION:**

Whether in children with Type1 DM, professional CGM along with SMBG decreases HbA1c significantly over 4months when compared to SMBG alone?

**Hypothesis:** Professional CGM when combined with SMBG, improves HbA1c in Type1 diabetic children (2 to 8 years old) when compared to SMBG alone.

**MATERIAL AND METHODS**

**Setting**: Pediatric Diabetic clinic, Advanced Pediatrics Centre, Postgraduate Institute of Medical Education and Research, Chandigarh.

**Study Period**: July 2014 to Dec 2015

**Study Design:** Prospective**,** Open Label Randomized control trial.

**Subjects**:

Children with Type 1 Diabetes attending our Pediatric Diabetes Clinic, between the age group of 2-10 yrs, will be randomized into two groups; Professional CGM plus SMBG (intervention group/Cases) and SMBG alone (control group).

**Inclusion criteria**:

1. Children with a diagnosis of Type 1 DM for at least 6 month
2. Age between 2 to 10 years.

**Exclusion Criteria**:

1. Children with history of acute metabolic decompensation such as DKA within previous 2 months.
2. Children on chronic medications known to affect blood glucose such as systemic corticosteroids.
3. Patients with known poor compliance and HbA1c >12%.
4. Associated co-morbidities like Celiac disease and Hypothyroidism.

**Randomization and allocation concealment:** Participants will be randomized to intervention (Professional CGMS+SMBG) or control (SMBG alone) arms in equal numbers. Randomization will be done using computer generated random number list. Group allocation will be concealed with opaque sealed envelopes.

**Sample Size**: With a 1:1 randomization ratio and based on the assumption of a common SD of 1.5%, an absolute difference of 1% in HbA1c between study groups can be detected with an alpha error of 0.05 (two sided) and a beta error of 0.20, using formula n= 2 x K x SD^2^ /delta ^2^ (where K is constant, SD is standard deviation and delta is magnitude of difference to be detected). Sample size of 34 participants each in the intervention and control group will be required.

**Statistical analysis:**Baseline characteristics will be calculated as means and SD. Results will be presented as means (95% CI).Statistical significance will be set at P<0.05. The primary endpoint will be the unit change in HbA1c over 4 months from intervention. Secondary end points will be percentages of low (<70) and high (>200) sugar records of the total sugar records (SMBG). Continuous variables will be compared using a two-sided *t* test or paired *t* test for change within each subject/group before and after intervention (Professional CGM). Intergroup comparisons of HbA1c will be made using a signed-rank test. Univariate ANOVA will be used to examine factors that may confound the change in HbA1C. Data will be analysed using SPSS (SPSS Inc., Chicago, IL, version 18.0 for Windows).

**Definition of Hypoglycemia**

The following definitions will be used for hypoglycemic events: *asymptomatic hypoglycemia* BG <50 mg/dL without symptoms suggestive of hypoglycemia; *symptomatic hypoglycemia* as suggestive clinical symptoms (headache, tremors, sweating, abdominal pain, mood changes) confirmed by blood glucose <50 mg/dL; *severe symptomatic hypoglycemia* as an event with severe symptoms (unconsciousness, use of intravenous glucose or requiring the assistance of another person) with either a blood glucose level <50 mg/dL or prompt recovery after administration of oral carbohydrates, intravenous glucose or glucagon; *nocturnal symptomatic hypoglycemia* as symptomatic hypoglycemia occurring during sleep between bedtime and rising in the morning or before the fasting blood glucose measurement and morning insulin injection.

**Study Procedures:**

**Intervention group:** All children enrolled in the intervention group will be placed on CGM (*i* Pro 2 Professional CGM™) for 72 hours and finger-prick blood glucose will also be monitored by glucometer (SMBG) at least 4 times per day with proper record of time. CGMS will be placed, on each patient enrolled after written informed consent, by investigator (after proper training) under supervision of the Physician In-charge. These patients will also record their daily activity, meals, snacks and other relevant information with exact time. Record of timing and doses of insulin injections will be maintained. All above information will be recorded on a patient log sheet by caregiver. Information with respect to actual duration and timing of wearing of CGM, any problems occurring in wearing CGM (like proper fitting, local irritation or redness/ pain), reasons for removing CGM if at all, any restriction in daily activities of the child (as told by parents/child) will be recorded,. Patients will continue to do SMBG during and after CGM. CGMS will be removed after 72 hours by the investigator. Report will be prepared using online Therapy Management Software after connecting CGMS with computer. Analysis of Professional CGM report will be done by one of the two Clinicians In-charge of the clinic and further modification in treatment will be advised based on CGM and SMBG records. Also investigations done prior (auto-immune workup, HbA1c, thyroid function test, TTG IgA etc) will be recorded. Mean of atleast 2 previous HbA1c (excluding one at diagnosis) will be recorded, Percentage of Low and high blood sugars recorded over previous 3 months and 3 months post CGM will be recorded. After modification of treatment guided by CGMS and SMBG, these patients will be followed every 2 months (for at least 4 months) with HbA1c levels and SMBG recorded on Diabetic Log book/Diary. Average of HbA1c on 2 occasions post CGM will be recorded. Also percentage of low sugar records seen on SMBG will be noted in follow up.

**Control group**: All other procedures will be same except that, no Professional CGM will be done in this group and treatment advice/modification will be based only on SMBG records.

On each visit patients in both groups will get diabetic education regarding importance of glucose control, insulin dose titration, diet management and exercise etc by same set of clinician and dietician.

**Logistic requirements for the study:**

Professional CGM device, sensors, software, dock, cables and chargers for downloading, Computer (to download data), Color printer (ideal, but not mandatory, to print data) and glucometer.

These are all available in the unit, except for sensors, which are available but less than the number of patients to be enrolled. So, around 15 sensors will have to be procured.

**Ethical Justification**

The study will be undertaken after the ethical clearance from the Institute’s Ethical Committee and after registering with clinical trial registry. Written informed consent will be taken from the parents prior to the participation in the study. The study involves a minimally invasive procedure (placement of Professional CGM subcutaneous, on the abdominal wall), for intervention group, for which consent will be taken. There are only minimal local side effects (itching, redness, swelling etc) reported with placement of CGM and in case of these side effects CGM will be immediately removed. Placement of the device does not hamper daily activities of the child; rather child will be encouraged to do his routine activities as before, to have maximum benefit of CGM. Participation in this study is expected to improve treatment of the child. In fact, identifying the hypoglycemic episodes would help in the better management and tight glycemic control of the child which prevents long-term complications. This knowledge would also help in designing better educational and intervention programs for children with Type 1 DM. Patients in the control arm are also expected to be benefitted from the study by more close monitoring, diabetic education and close supervision of the SMBG records. Cost of Professional CGM will be borne by the investigators. No extra blood tests or treatment will be provided to study groups.

**BIBLIOGRAPHY**

1. Thomas C. Blevins, Bruce W. Bode, Satish K. Garg, George Grunberger, Irl B. Hirsch, Lois Jovanovič et al ***,***AACE Consensus statement , continuous glucose monitoring , Endocrpract 2010 ; 16(5) 732.
2. Thomas C. Blevins.Professional Continuous Glucose Monitoring in Clinical Practice 2010.*Diabetes SciTechnol*2010 4: 440
3. The Diabetes Control and Complications Trial Research Group. The effect of intensive treatment of diabetes on the development and progression of long-term complications in insulin-dependent diabetes mellitus. N Engl J Med 1993;329: 977–986.
4. Yates K, Hasnat Milton A, Dear K, Ambler G. Continuous glucose monitoring‑guidedinsulin adjustment in children and adolescents on near‑physiological insulin regimens: a randomized controlled trial. Diabetes Care. 2006;29: 1512-1517.
5. Langendam M, Luijf YM, Hooft L, Devries JH, Mudde AH, Scholten RJ. Continuous glucose monitoring systems for type 1 diabetes mellitus.Cochrane Database SystRev. 2012 Jan 18;1:CD008101.
6. Szypowska A, Ramotowska A, Dzygalo K, Golicki D. Beneficial effect ofreal-time continuous glucose monitoring system on glycemic control in type 1diabetic patients: systematic review and meta-analysis of randomized trials. EurJEndocrinol. 2012 Apr;166(4):567-74
7. Golden SH, Brown T, Yeh HC, Maruthur N, Ranasinghe P, Berger Z et al. Methods for Insulin Delivery and Glucose Monitoring: Comparative Effectiveness [Internet]. Rockville (MD): Agency for HealthcareResearch and Quality (US); 2012 Jul. Available from <http://www.ncbi.nlm.nih.gov/books/NBK99217>.
8. Lagarde WH, Barrows FP, Davenport ML, et al. Continuous subcutaneousglucose monitoring in children with type 1 diabetes mellitus: a single‑blind,randomized, controlled trial. Pediatr Diabetes. 2006; 7: 159-164.
9. Deiss D, Hartmann R, Schmidt J, Kordonouri O. Results of a randomized controlled cross‑over trial on the effect of continous subcutaneous glucosemonitoring (CGMS) on glycaemic control in children and adolescents withtype 1 diabetes. ExpClinEndocrinol Diabetes.2006; 114: 63-67.
10. Ludvigsson J, Hanas R. Continuous subcutaneous glucose monitoringimprovedmetaboliccontrol in pediatric patients with type 1 diabetes: a controlledcrossover study. Pediatrics.2003; 111: 933-938.
11. Chase HP, Beck RW, Xing D, Tamborlane WV, Coffey J, Fox LA, et al. Continuous glucose monitoring in youth with type 1 diabetes: 12-month follow-up of the Juvenile Diabetes Research Foundation continuous glucose monitoring randomized trial. Diabetes TechnolTher. 2010 Jul;12(7):507-15.
12. [Elsie M. Taveras](http://www.sciencedirect.com/science/article/pii/S1551714412002327),[RichardMarshall](http://www.sciencedirect.com/science/article/pii/S1551714412002327)[^c^](http://www.sciencedirect.com/science/article/pii/S1551714412002327#af0015), [Christine M. Horan](http://www.sciencedirect.com/science/article/pii/S1551714412002327) ,[Matthew W. Gillman](http://www.sciencedirect.com/science/article/pii/S1551714412002327), [Karen Hacker](http://www.sciencedirect.com/science/article/pii/S1551714412002327), [Ken P. Kleinman](http://www.sciencedirect.com/science/article/pii/S1551714412002327)et al contemporary clinical trials[Volume 34, Issue 1](http://www.sciencedirect.com/science/journal/15517144/34/1), January 2013, Pages 101–108.
13. Juvenile Diabetes Research Foundation Continuous Glucose Monitoring Study Group, Bode B, Beck RW,et al. Sustained benefit of continuous glucose monitoringon A1C, glucose profiles, and hypoglycemia in adults withtype1 diabetes. *Diabetes Care*. 2009;32:2047-2049.
14. Diabetes Research in Children Network (DirecNet) Study Group, Buckingham B, Beck RW, et al. Continuous glucose monitoring in children with type 1 diabetes.*JPediatr*. 2007;151:388-393.
15. Jovanovič L. The role of continuous glucose monitoring in gestational diabetes mellitus.*DiabetesTechnolTher*. 2000;2(Suppl 1):S67-S71.
16. Yogev Y, Chen R, Ben-Haroush A, Phillip M, Jovanovič L, Hod M. Continuous glucose monitoring for the evaluationof gravid women with type 1 diabetes mellitus. *ObstetGynecol*. 2003;101:633-638.
17. Chen R, Yogev Y, Ben-Haroush A, Jovanovič L, Hod M, Phillip M. Continuous glucose monitoring for the evaluation and improved control of gestational diabetes mellitus.*JMaternFetal Neonatal Med*. 2003;14:256-260.
18. Murphy HR, Rayman G, Lewis K, et al. Effectiveness of continuous glucose monitoring in pregnant women with diabetes: randomised clinical trial. *BMJ*. 2008;337:a1680.
19. Langendam M, Luijf YM, Hooft L, Devries JH, Mudde AH, Scholten RJ. Continuous glucose monitoring systems for type 1 diabetes mellitus. Cochrane Database Syst Rev. 2012 Jan 18;1:CD008101.
20. Szypowska A, Ramotowska A, Dzygalo K, Golicki D. Beneficial effect of real-time continuous glucose monitoring system on glycemic control in type 1 diabetic patients: systematic review and meta-analysis of randomized trials. Eur J Endocrinol. 2012 Apr;166(4):567-74.
21. Golden SH, Brown T, Yeh HC, MaruthurN, Ranasinghe P, Berger Z et al. Methods for Insulin Delivery and Glucose Monitoring: Comparative Effectiveness [Internet]. Rockville (MD): Agency for Healthcare Research and Quality (US); 2012 Jul. Available from <http://www.ncbi.nlm.nih.gov/books/NBK99217/>
22. Yeh HC, Brown TT, Maruthur N, Ranasinghe P, Berger Z, Suh YD et al. Comparative Effectiveness and Safety of Methods of Insulin Delivery and Glucose Monitoring for Diabetes Mellitus: A Systematic Review and Meta-analysis. Ann Intern Med. 2012 Jul 10:E-508.
23. Pickup JC, Freeman SC, Sutton AJ. Glycaemic control in type 1 diabetes during real time continuous glucose monitoring compared with self monitoring of blood glucose: meta-analysis of randomised controlled trials using individual patient data. BMJ 2011;343:d3805
24. Gandhi GY, Kovalaske M, Kudva Y, Walsh K, Elamin MB, Beers M, Coyle C, Goalen M, Murad MS, Erwin PJ, Corpus J, Montori VM, Murad MH. Efficacy of continuous glucose monitoring in improving glycemic control and reducing hypoglycemia: a systematic review and meta-analysis of randomized trials. J Diabetes Sci Technol. 2011 Jul 1;5(4):952-65.

**PATIENT PROFORMA**

Patient Name: Age/ DOB: Sex

CR/Adm No:

Address and Phone #:

Date and time of starting CGM:

Date and time of completing CGM:

**Date of diagnosis of Type1 DM:**

**Current Therapy:** MDI – Daily no. of injections: 2/3/4.

Time for injections:

**Insulin regimen:** NPH Lantus/DetemirHumalog/Novolog/ApidraRegular

Mixed Insulin Type

**Current SMBG**: NO: 2/3/4 per day Timings:

Current glucose control: HbA1c over last 6 months-1 year:

Initial workup:

Antibody assays: GAD 65/ ICA

TTG Ig A

Thyroid function test:

**Patient log sheet while on CGMS:-**

1. Blood Glucose (4 times, pre-meals And bed time) with exact time.
2. Insulin doses with time.
3. Details of Meals and Snacks.
4. Significant physical activity (time/duration).
5. Reason for premature removal of device (if at all).
6. Accidental removal (if at all).
7. Any problems perceived by patient: Local Site: Pain/ Redness/irritation/swelling/ others. Problem of fixing/stability of CGM device. Any restriction of routine activities.
8. Any problems perceived by parent: Anxiety/ apprehension/fears. Sleep disturbance (parent/child) and Others.

**FINDINGS OF CGMS REPORT:**

1. Nocturnal Hypoglycemia.
2. Nocturnal Hyperglycemia.
3. Rebound hyperglycemia / Somoygi effect.
4. Dawn phenomenon.
5. Pre-meal Hypoglycemia/ hyperglycemia.
6. Post-meal Hypoglycemia/ Hyperglycemia.

**Treatment changes advised after analysis of CGMS and SMBG or SMBG alone:**

|  | **CGMS+SMBG** | **SMBG ALONE** |
| --- | --- | --- |
| *1*)  change in one or more bolus or rapidacting  insulin dosage; *2*) change in one or  more basal or intermediate/long-acting  insulin dosage; *3*) change in the correction  algorithm (3); *4*) alteration in the approach  to exercise to include change in  carbohydrate management (11); *5*) reinforcing  treatment algorithm for hypoglycemia  to include rechecking, adding extra  carbohydrate, and protein (11); *6*) change  in early morning basal rate (pump patients)  or bedtime insulin dosage and/or  timing (injection patients) due to the  dawn phenomenon; *7*) increase in meal  bolus and timing of injection after the  meal (injection patients) or adding  square-wave bolus (pump patients) for  high-fat meals; *8*) increase in meal insulin  dosage for high glycemic foods; and *9*)  referral for counseling to improve adherence  with diabetes regimen. |  |  |

**Parameters before and after intervention (POST-CGMS and SMBG Vs SMBG alone):**

|  | **CGMS+SMBG** | | | **SMBG ALONE** | | |
| --- | --- | --- | --- | --- | --- | --- |
|  | **Base line** | **2 months** | **4 months** | **Base line** | **2 months** | **4 months** |
| **HbA1c** |  |  |  |  |  |  |
| Percentage of low blood sugar |  |  |  |  |  |  |
| Percentage of high blood sugar |  |  |  |  |  |  |
|  |  |  |  |  |  |  |
|  |  |  |  |  |  |  |

**Participants Information sheet**

INVESTIGATORS: Dr.Ravi Teja(PI).

Guide: Dr.Rakesh Kumar

Co-Guides: 1. Dr. Devi Dayal 2. Dr.Naresh Sachdeva

Name of Participant: …………………..

**Title of study**: Professional Continuous Glucose Monitoring In Children With Type 1 Diabetes Mellitus: An Open Label Randomised Control Trial

You (your child) are invited to take part in this research study. The information in this document is meant to help you decide whether or not to take part. Please feel free to ask if you have any queries or concerns.

You (your child) are being asked to participate in this study being conducted in APC, PGIMER, CHANDIGARH because you satisfy our eligibility criteria which are:

(1) Diagnosis of Type1 Diabetes for more than 6 months.

(2) Age less than 8 years

You (your child) will be one of the 70 patients we plan to recruit in this study.

**What is the purpose of research?**

Continuous glucose monitoring (CGM) has long been used and proven to be effective in achieving target sugar and HbA1c levels in patients with type 1 diabetes. However, prohibiting cost of CGM devices has limited its use in developing and low income countries like ours. Children with type 1 diabetes are thus advised self monitoring of blood sugar (SMBG) using finger prick and traditional glucose meters. With the result there is scarcity of information on use of CGM devices in Indian children with type 1 diabetes. We have planned to conduct a pilot study for assessing performance of one of these CGM devices in hospitalized children with type 1 diabetes. The CGM device and all other logistics required will be provided by us.We have obtained permission from the Institutional Ethics Committee for conducting this study.

**The study design-**open label randomized controlled trial.

**Study Procedures-**All children enrolled in the study will be put on CGM for 72 hours and finger-prick blood glucose will also be monitored by glucometer at least 4 times per day with proper record of time. All information will be recorded on a proforma with respect to actual duration and timing of wearing of CGM, any problems occurring in wearing CGM (like proper fitting, local irritation or redness/ pain), reasons for removing CGM prematurely, all meals and snacks taken with time for those 72 hours of monitoring. While on CGM device, patient/ caregiver will have to keep record of all meals, blood sugars (at least 4 /day), exercise, symptoms of low/high sugars and any other event which you feel could affect sugar level of the child. Also investigations done prior (auto-immune workup, HbA1c, thyroid function test, TTG IgA etc) will be recorded. Parents as well as patient’s views regarding convenience and difficulties in keeping CGM on for long time will be recorded. What problems are encountered by patients/parents in performing daily activities with CGM on will also be recorded.

**Possible risks to you-**Your child is not being exposed to any new drug or treatment. The CGM is standard of care for type 1 diabetes patients in developed world. The CGM requires the insertion of a glucose sensor into the skin. Adverse events associated with glucose sensor insertion are highly uncommon and are limited to bleeding, irritation, pain, rash, infection, raised bump, and irritation at the site from the tape or bandage to secure the CGM to the skin.

**Possible benefits to you-**The CGM records glucose values in the interstitial fluid continuously (as against 3-4 times/day which you are already doing) and allows your doctor to review glucose patterns and trends which will help in deciding appropriate insulin dosage and regimen. Also any asymptomatic hypoglycemia occurring in those 72 hours can be detected. In the long run this testing may help improve better sugar control and lower HbA1c levels.

**Possible benefits to other people-**The results of the research may provide benefits to the society in terms of advancement of medical knowledge and/or therapeutic benefit to future patients.

**Cost to the participant-** There is no extra cost incurred to the patient after enrolling in study. CGM device and glucose sensor required for this study will be provided free.

**Who is paying for this research?**

The PGIMER is sponsoring the cost of glucose sensor and other accessories required.

**What should you do in case of injury or a medical problem during this research study?**

Your safety is the prime concern of the research. If you are injured or have a medical problem as a result of being in this study, you should contact one of the people listed at the end of the consent form. You will be provided the required care/treatment.

**Confidentiality of the information obtained from you-**You have the right to confidentiality regarding the privacy of your medical information (personal details, results of physical examinations, investigations, and your medical history). By signing this document, you will be allowing the research team investigators, other study personnel, sponsors, institutional ethics committee and any person or agency required by law. The results of clinical tests and therapy performed as part of this research may be included in your medical record. The information from this study, if published in scientific journals or presented at scientific meetings, will not reveal your identity.

**How will your decision to not participate in the study affect you?**

Your decision not to participate in this research study will **not affect your medical care or your relationship with the investigator or the institution**. Your doctor will still take care of you and you will not lose any benefits to which you are entitled.

**Can you decide to stop participating in the study once you start?**

The participation in this research is purely voluntary and you have the right to withdraw from this study at any time during the course of the study without giving any reasons. However, it is advisable that you talk to the research team prior to stopping the treatment. You may be advised about how best to stop the treatment safely. If you withdraw, you may be asked to undergo some additional tests to which you may or may not agree. Though advisable that you give the investigators the reason for withdrawing, it is not mandatory.

**Can the investigator take you off the study?**

You may be taken off the study without your consent if you do not follow instructions of the investigators or the research team or if the investigator thinks that further participation may cause you harm.

**Right to new information-** If the research team gets any new information during this research study that may affect your decision to continue participating in the study, or may raise some doubts, you will be told about that information.

**Patient/parent Instructions:**

• Continuously wear the Recorder and glucose sensor.

• Complete the Patient Log Sheet daily as instructed.

• Test blood sugar 4 times a day using a BG meter.

• Protect the Recorder and glucose sensor site from accidental removal.

• Keep the tape and/or bandage intact on your skin.

• Do not administer insulin close to the glucose sensor site.

**Contact persons-** For further information / questions, you can contact us at the following address:

**Principal Investigator: :**Dr.K.V.RaviTeja, Junior Resident, Dept. of Pediatrics, APC, PGIMER, Chandigarh. Ph: 8195862345.

**Guide:** Dr. Rakesh Kumar, Asstt. Professor, Dept. of Pediatrics, APC, PGIMER, Chandigarh. Ph: 9914208480

**Co-Guides:** Dr Devi Dayal, Associate Professor, Dept. of Pediatrics, APC,, PGIMER, Chandigarh.

In case of conflicts, you can contact the chairperson (convener) of our institutional ethics committee.

**Patient consent form**

Patient Name: Age/ DOB: Sex CR/Adm No:

Address and Phone #:

**Title of the study**: Professional CGM in children with type 1 DM open label randomised controlled trial.

**Name of the Principal /(Co-) Investigator**: Dr. K V Ravi Teja/ Dr.Rakesh Kumar/Dr. Devi Dayal.

**Name of the Institution**: Deptt of Pediatrics, APC, PGIMER, Chandigarh.

**Name and address of the sponsoring (funding) agency** : PGIMER, Chandigarh

**Documentation of the informed consent**

I, … … … … … … … … … ., have read the information in this form (or it has been read to me). I was free to ask any questions and they have been answered. I am over 18 years of age and, exercising my free power of choice, hereby give my consent for my child to be included as a participant in “Performance assessment of the Continuous Glucose Monitoring System and its use for measurement of glucose trends in children with Type 1 diabetes.”

(1) I have read and understood this consent form and the information provided to me.

(2) I have had the consent document explained to me.

(3) I have been explained about the nature of the study.

(4) My rights and responsibilities have been explained to me by the investigator.

(5) I have been advised about the risks associated with my participation in the study.

(6) I agree to cooperate with the investigator and I will inform him/her immediately if I suffer unusual symptoms.

(7) I have not participated in any research study within the past **… ..month(s).**

(8) I am aware of the fact that I can opt out of the study at any time without having to give any reason and this will not affect my future treatment in the hospital.

(9) I am also aware that the investigators may terminate my participation in the study at any time, for any reason, without my consent.

(10) I hereby give permission to the investigators to release the information obtained from me as result of participation in this study to the sponsors, regulatory authorities, Government agencies, and ethics committee. I understand that they may inspect my original records.

(11) My identity will be kept confidential if my data are publicly presented.

(12) I have had my questions answered to my satisfaction.

(13) I have decided to be in the research study.

I am aware, that if I have any questions during this study, I should contact at one of the addresses listed above. By signing this consent from, I attest that the information given in this document. I will be given a copy of this consent document.

Whether **child’s assent** was asked: **Yes/ No** (Tick one) [If the answer to the above question is Yes, write the following phrase: You agree with the manner in which assent was asked for from your child and given by your child. You agree to have your child take part in this study.] [If answer to the above question is No, give reason(s):

Although your child did not or could not give his or her assent, you agree to your child’s participation in this study.

Name and signature / thumb impression of the **participant’s parent(s) (or legal representative**):

_________________ (Name) ___________ (Signature) Date: __________ Time: ________

Name and signature of impartial **witness** (required if parents of participant child illiterate):

__________________ (Name) ___________ (Signature) Date:__________ Time: _________

Address and contact number of the impartial witness____________________________________________

Name and signature of the **Investigator or his representative obtaining consent**:____________________ (Name) _________________ (Signature) ____________________ (Date)

**Consent Form for Professional CGMS Recording**

**Title of Study:**Professional CGM in children with type 1 DM open label randomised controlled trial

**Investigators:**

Dr.K.V.Ravi Teja (PI).

Guide - Dr.Rakesh Kumar.

Co-Guides: Dr. Devi Dayal / Dr.Naresh Sachdeva

Dear Patient/Parent/Gaurdian,

Please read this Patient Consent Form carefully, as you will be asked to provide your agreement and consent to the terms and instructions below regarding your use of the digital recorder, a continuous glucose monitor, and this office’s use of the web-based application for your diabetes therapy. If there is anything in this Consent Form that you do not understand or have concerns with, please let us know. Please note that if you do not agree to the terms of this Consent Form, our office cannot place a glucose sensor on you, nor use the application for your diabetes treatment.

This Consent Form is separate from, and in addition to, any other consent or authorization form you have received from us.

**What is Professional continuous glucose monitoring and how it is done?**

This is a continuous glucose monitor placed on your body (over abdomen) for a period of a 3-7 days, that will continuously record your glucose levels. The results of the recordings can be recorded, placed in a report, analyzed, etc., and will assist us in helping you manage your diabetes more effectively. This should only be used pursuant to our instructions. When wearing this, you agree and commit to the following:

(a) You agree to collect at least 4 blood glucose finger-stick tests per day.

(b) You agree to enter all meals, medication, and other relevant activities on a log sheet.

(c) You agree to check the insertion site daily, to verify the sensor is fully inserted and that the site is not irritated, excessively red or painful. (You understand that the possible risks include inflammation, infection, and/or bleeding at the sensor insertion site.)

(d) You agree to return the device within __________ days of completing the wear period.

(e) You agree to take every precaution when handling the device , and understand it is a sensitive medical device. You may be responsible for the loss or theft, or any damage or malfunction of device caused by any unreasonable or unusual activity.

(f) In addition, you agree to follow any other specific instructions we provide , and to call us immediately if you experience problems or have questions.

If you have any questions, please contact us or our colleague.

By signing below, I acknowledge that I have read, fully understand, and agree to the above terms of this Consent Form, including those terms regarding the use of CGM device and the storage and use of my Protected Information (as described above) through the website. I have had an opportunity to ask questions and to receive answers. I realize that my consent is voluntary, and I may refuse to participate or utilize the benefits.

­­­___________________________________ _____________________

Signature of Patient (or Legal Representative) Date

___________________________________

Print Patient Name

If you would like any further information, or have any further queries concerning the research study, you are encouraged to contact the research team.

**Dr. K.V. Ravi Teja Principal Investigator, Junior Resident**

**Department of Pediatrics, PGIMER, Chandigarh**

**E-mail:** [**kvravitheja@gmail.com**](mailto:kvravitheja@gmail.com)

**Phone no: 8195862345.**
