# Supplementary material for: Clinical efficacy of Professional Continuous Glucose Monitoring in improving glycemic control among children with Type 1 Diabetes Mellitus: An Open-label Randomized Control Trial
Source: Sci Rep. 2019 Apr 16;9:6120. doi: 10.1038/s41598-019-42555-6 (PMC6467868; doi:10.1038/s41598-019-42555-6)
Supplement: Supplementary file 2 — Registration of Trial [file 41598_2019_42555_MOESM2_ESM.pdf]

Clinical Trial Details (PDF Generation Date :- Thu, 24 Jan 2019 07:20:38 GMT)

**CTRI Number**	CTRI/2017/02/007937 [Registered on: 21/02/2017] - **Trial Registered Retrospectively**
**Last Modified On**	15/02/2017
**Post Graduate Thesis**	Yes
**Type of Trial**	Interventional
**Type of Study**	Medical Device
**Study Design**	Randomized, Parallel Group Trial
**Public Title of Study**	Professional Continuous Glucose Monitoring versus Self-Monitoring of Blood Glucose in children with Type 1 Diabetes Mellitus
**Scientific Title of Study**	Professional Continuous Glucose Monitoring and self monitoring of blood glucose in children with Type 1 Diabetes Mellitus: an Open Label Randomized Control Trial

Secondary IDs if Any

Secondary ID	Identifier
NIL	NIL

Details of Principal Investigator or overall Trial Coordinator (multi-center study)

Details of Principal Investigator	
**Name**	Dr Rakesh Kumar
**Designation**	Assoc. Professor
**Affiliation**	PGIMER,, Chandigarh
**Address**	Deptt of Pediatrics, PGIMER, Sector 12,Chandigarh<br>Chandigarh<br>CHANDIGARH<br>160012<br>India
**Phone**	
**Fax**	
**Email**	drrakesh.pgi@gmail.com

Details Contact Person (Scientific Query)

Details Contact Person (Scientific Query)	
**Name**	Dr KV Ravi Teja
**Designation**	PG student, Pediatrics
**Affiliation**	PGIMER,, Chandigarh
**Address**	Deptt of Pediatrics, PGIMER, Sector 12,Chandigarh<br>Chandigarh<br>CHANDIGARH<br>160012<br>India
**Phone**	91-8195862345
**Fax**	
**Email**	kvravitheja@gmail.com

Details Contact Person (Public Query)

Details Contact Person (Public Query)	
**Name**	Dr Rakesh Kumar
**Designation**	Assoc. Professor
**Affiliation**	PGIMER,, Chandigarh
**Address**	Deptt of Pediatrics, PGIMER, Sector 12,Chandigarh<br>Chandigarh<br>CHANDIGARH<br>160012<br>India
**Phone**	
**Fax**	
**Email**	drrakesh.pgi@gmail.com

Source of Monetary or Material Support

Source of Monetary or Material Support
> Institute Thesis Grant through Head Of Deptt., Deptt of Pediatrics, APC Building Level 3A, PGIMER, Chandigarh. 160012

Primary Sponsor

Primary Sponsor Details	
**Name**	PGIMER Chandigarh
**Address**	Director, PGIMER, Chandigarh, Sector 12, Chandigahr UT 160012
**Type of Sponsor**	Research institution and hospital

Details of Secondary Sponsor

Name	Address
NIL	NIL

Countries of Recruitment

List of Countries
India

Sites of Study

Name of Principal Investigator	Name of Site	Site Address	Phone/Fax/Email
Dr Rakesh Kumar	PGIMER, Chandigarh	Room No 3117, Level 3A,Deptt of Pediatrics,APC Building Chandigarh CHANDIGARH	7087008480<br><br>drrakesh.pgi@gmail.com

Details of Ethics Committee

Name of Committee	Approval Status	Date of Approval	Is Independent Ethics Committee?
Institute Ethic Committee (Intramural), PGIMER, Chandigarh	Approved	03/09/2014	No

Regulatory Clearance Status from DCGI

Status	Date
Not Applicable	No Date Specified

Health Condition / Problems Studied

Health Type	Condition
Patients	Type 1 Diabetes Mellitus

Intervention / Comparator Agent

Type	Name	Details
Comparator Agent	Self monitoring of blood glucose (SMBG) by glucometer	Treatment is guided /modified only on basis of SMBG by patients
Intervention	Professional Continous Glucose montoring (CGM) by a device for 3-5days PLUS Self monitoring of Blood Glucose (SMBG) by glucometer	Treatment is guided/modified by result of Professional CGM AND SMBG both. SMBG will be continued as in comparator and CGM will be done for 3-5 days.

Inclusion Criteria

Inclusion Criteria	
**Age From**	2.00 Year(s)
**Age To**	10.00 Year(s)
**Gender**	Both
**Details**	1. Children with a diagnosis of Type 1 DM for at least 6 month<br>2. Age between 2 to 10 years.<br>3. Patients on basal-bolus regimen with insulin analogues with 3-4 injections per day and doing SMBG at least 3-4 times per day.

Exclusion Criteria

Exclusion Criteria	
**Details**	1. Children with history of acute metabolic decompensation such as DKA within previous 2 months.<br>2. Children on chronic medications known to affect blood glucose such as systemic corticosteroids.

		3. Patients with known poor compliance and HbA1c >12%.

**Method of Generating Random Sequence**	Computer generated randomization
**Method of Concealment**	Sequentially numbered, sealed, opaque envelopes
**Blinding/Masking**	Open Label

Primary Outcome

Outcome	Timepoints
Unit Change in HbA1c values	3 months post intervention

Secondary Outcome

Outcome	Timepoints
Frequency of Low and high blood sugar records	3 months post treatment

**Target Sample Size**	**Total Sample Size=**70<br>**Sample Size from India=**70
**Phase of Trial**	N/A
**Date of First Enrollment (India)**	14/02/2015
**Date of First Enrollment (Global)**	No Date Specified
**Estimated Duration of Trial**	**Years=**0<br>**Months=**9<br>**Days=**0
**Recruitment Status of Trial (Global)**	Not Applicable
**Recruitment Status of Trial (India)**	Completed
**Publication Details**	none

Brief Summary

Primary objective:

1. To assess efficacy of treatment modulation based on professional CGM (done over 3 days) in improving glycemic control compared to self-monitoring blood glucose.

Secondary Objectives:

1. To assess feasibility and acceptability of professional CGM in diabetic children between 2 to 10 years of age.

2. Efficacy of professional CGM in reducing hypoglycemic and hyperglycemic episodes.

RESEARCH QUESTION:

	Whether in children with Type1 DM, professional CGM along with SMBG decreases HbA1c significantly over 3-4 months when compared to SMBG alone?<br><br>**Hypothesis:** Professional CGM when combined with SMBG, improves HbA1c in Type1 diabetic children (2 to 10 years old) when compared to SMBG alone.
